# Protein stereo‐folding and proteostasis: A new path for clinical and translational medicine

**DOI:** 10.1002/ctm2.70719

**Published:** 2026-07-14

**Authors:** Yuhan Wang, Yuyang Qiu, Xiangdong Wang

**Affiliations:** ^1^ Department of Pulmonary and Critical Care Medicine, Zhongshan Hospital Fudan University Shanghai Medical College Shanghai China; ^2^ Shanghai Institute of Clinical Bioinformatics Shanghai China; ^3^ Fudan University Center of Clinical Bioinformatics Shanghai China

Proteins are fundamental biochemical elements in the body and play important roles in maintaining the biological functions of cells and organs. Protein homeostasis (proteostasis) within intra‐ and extracellular microenvironments promotes molecular communication among organs, cells, and organelles. The nature, content, network, and spatiotemporal dynamics of extracellular proteostasis, including chaperones, proteolytic machineries, and the regulators, change during the development of cellular and organ dysfunction, tumourigenesis, inflammation, and aging.^1^, ^2^ Among these alterations, the spatial shifting and remodelling of extra‐ and intracellular proteostasis are highly dependent upon the evolution of para‐cellular microenvironmental functions and responses to systemic and local challenges. For example, altered proteostasis may reflect the microenvironment‐dependent microglial cell states and heterogeneity of myeloid cells in the human brain and identify the blood–brain barrier, meningeal components and cell–cell interactions in brain diseases. These changes can be measured by integrating the codetection by indexing with the high‐parameter capabilities of single‐cell technologies with spatial resolution in brain tissue.^3^ Spatial proteostasis maintains stereological spatialisation and interactions among stereo‐cells and organelles and represents an important component of stereo‐cell biomedicine,^4^, ^5^ a new approach advancing the identification and development of disease‐specific biomarkers and therapeutic targets,^4^, ^5^, ^6^ and an innovative pathway towards next generation clinical translation and precision medicine.^7^ **^,^** ^8^ The current Commentary highlights the importance of protein stereo‐folding and proteostasis as components of the intra‐ and extracellular microenvironment that maintain optimal bio‐ecosystems for cell and organelle interactions. Through artificial intelligent (AI) system analyses and virtual screening, an efficient and accurate protein structure prediction modelling developed is accelerating the translational progress from protein biology to drug discovery and to novel therapies.

The dynamic changes in protein stereo‐folding and structure determine the biochemical nature and function of proteostasis. Mutations in amino acids or abnormal alterations in stereo‐folding along the protein chain can cause structural instability and dysfunction, representing critical factors that influence the quality of proteostasis. These elements highlight a dynamic protein folding continuum shaped by the microenvironment (Figure 1A). The significance of protein stereo‐folding in clinical and translational medicine remains underestimated and further exploration is required, as the complex three‐dimensional structure of individual proteins has historically been difficult to be characterised because of challenges in identifying stereo‐folding patterns. With the rapid development of biotechnologies, AI systems like AlphaFold can predict millions of protein 3D structures using their amino acid sequences with high accuracy and efficiency. Through continuously updated and improved systems, the stereological structures of proteins and their interactions within proteostasis, as well as the structures of molecular complexes (e.g., proteins, nucleic acids, small molecules, ions, and modified residues), can be predicted and modelled with increasing accuracy. These include protein–ligand interactions, protein–nucleic acid interactions, antibody–antigen predictions, and biomolecular spatial connections.^9^ The stability and function of extra‐ and intracellular proteostasis, especially in three‐dimensional environments, are highly dependent upon the spatiotemporal configurations and localisation of molecules and their interactions. Multi‐dimensional analyses of the protein stereo‐folding and proteostasis enhance our understanding of stereological interactions among cells and organelles within extracellular and intracellular microenvironments. Consequently, moving beyond static predictions, precise spatial localisation within the stereo‐cell microenvironment can determine the functional and structure landscapes of protein stereo‐folding and proteostasis (Figure 1B).

**FIGURE 1:**
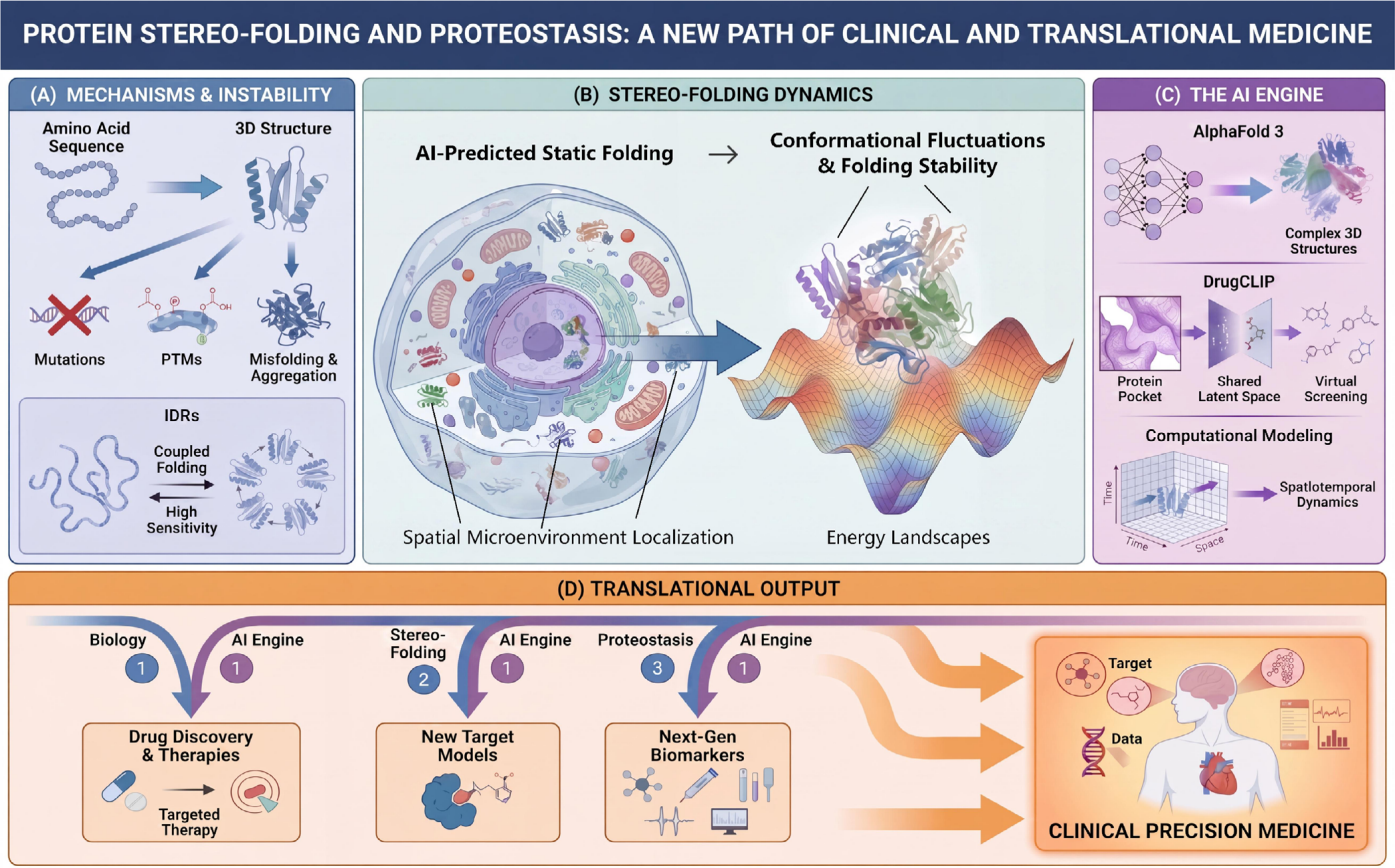
AI‐driven landscape of protein folding and proteostasis in clinical translation and precision medicine, to illustrate the integration of protein stereochemistry and homeostatic networks within the intra‐ and extracellular microenvironment, and the AI‐powered pipeline facilitating clinical translation. (A) The protein folding continuum from amino acid sequences to three‐dimensional structures highlights destabilising factors such as mutations, aberrant post‐translational modifications (PTMs), and misfolding or aggregation. Intrinsically disordered regions (IDRs) are highlighted for their high sensitivity to the microenvironment via coupled folding mechanisms. (B) Protein stereo‐folding within the stereo‐cell microenvironment. Moving beyond AI‐based static fold prediction (left), the stereo‐folding framework (right) highlights that the precise spatial localisation determines the protein energy landscapes. In this context, protein functional states are dictated by both conformational fluctuations and folding stability, in addition to static structures or expression contents. (C) Computational breakthroughs in structural biology and drug discovery. The AI‐based system like AlphaFold enables high‐precision structural prediction of complex biomolecular assemblies. The AI‐aided approach like DrugCLIP facilitates the high‐throughput virtual screening by mapping protein pockets and small molecules into a shared latent space. The multidimensional computational modelling elucidates the spatiotemporal dynamics of therapeutic targets. (D) The protein biology, structural biology, and AI‐accelerated analysis of protein stereo‐folding and proteostasis converge into three pillars of precision medicine: discovery of novel targeted therapies, development of robust target models, and identification of next‐generation biomarkers, ultimately advancing data‐driven, personalised clinical medicine.

Understanding of protein stereo‐folding and proteostasis also advances concepts in drug discovery and development. The protein targets‐based drugs are developed based on elucidating protein structures and their interactions using structural biology with techniques such as crystallography, cryoelectronic microscopy, drug‐target interactions at atomic levels, and deep neural networks utilising AI systems. For example, DrugCLIP was developed as a contrastive learning framework for fast and accurate virtual screening by encoding protein pockets and small molecules into a shared latent space.^10^ This represents a breakthrough in next‐generation drug discovery and development through the application of AI‐based, structure‐oriented screening supported by multilayer validation and highly efficient large‐scale screening capacity. Alongside tools like AlphaFold 3, these computational breakthroughs in multidimensional modelling are elucidating the spatiotemporal dynamics of therapeutic targets (Figure 1C). However, the stereo‐folding and spatial distributions of proteins and proteostasis introduce a new dimension to drug designing and targeting, ensuring therapeutic efficacy and targeting precision at stereological resolution. AI systems can accurately predict protein structure and folding from amino acid sequences, as well as protein–protein interactions, but they still have limited ability to predict the spatiotemporal localisation of proteins, the dynamic status of proteostasis, and the surrounding biological environment (bioecosystem) of target proteins. The nature of spatial proteostasis may affect drug targeting accuracy, binding and dislocation rate of drug interaction, and biochemical activation of drug functioning. Furthermore, the diversity, spatial organisation, and temporal dynamics of protein modifications add the substantial complexity to protein stereo‐folding and proteostasis in drug discovery and development. These modifications include phosphorylation, glycosylation, ubiquitination, acetylation, methylation, lipidation, hydroxylation, sumoylation, disulphide bond formation, proteolytic cleavage, nitrosylation, ADP‐ribosylation, carboxylation, sulphation, and deamidation.

Protein stereo‐folding and proteostasis may serve as novel sources for generating biology‐ and disease‐specific biomarkers, as well as therapeutic strategies, thereby reshaping conventional concepts and understanding. Folding complexities, dimensionality, and scaling directly or indirectly affect intramolecular binding and tractive forces, energy levels‐associated structures, biological functions, molecular interactions, misfolding‐caused polymerisation and aggregations, and the biochemical natures of proteins. Of those, the quality of protein stereo‐folding and proteostasis can be evaluated using large‐scale AI systems and physics‐based analyses of protein structure accuracy, energies of conformational fluctuations, and folding stability.^11^ The protein energy landscapes evidence that protein function is shaped not only by a static fold but also by conformational fluctuations and folding stability. It is therefore necessary to define those fluctuations and structural characteristics of proteins and develop novel strategies for biomarkers and target molecule identification. Intrinsically disordered regions (IDRs), protein segments that do not adopt stable three‐dimensional structures, mainly exist as highly dynamic and flexible ensembles of interconverting conformations with unique amino acid composition under physiological conditions. Some IDRs may undergo folding upon binding to partner molecules through a process known as coupled folding and binding, thereby increasing the sensitivity of protein stereo‐folding and proteostasis to intra‐ and extracellular environmental changes. With the development of AI systems and biological research, IDRs‐specific properties were characterised through integration of molecular grammars with NARDINI algorithm (Non‐random Arrangement of Residues in Disordered Regions Inferred using Numerical Intermixing).^12^ In addition, the protein pocket interactions‐based atom‐level model (PocketXMol) was proposed to be an AI‐aided drug discovery system for the structure prediction and design of small molecules (e.g., caspase‐9 inhibitors) and peptides (e.g., PD‐L1‐binders) with high specificities.^12^ The 4D whole‐cell spatial and kinetic model highlights the need to connect molecular structure with spatial location and time‐dependent cellular processes.^13^ These approaches facilitate links between protein structure and cellular biology, organelle biology, spatial organisation, protein‐specific functions and interactions, and proteostasis. These detailed features of protein and spatial structure will advance the discovery and development of the next generation biomarkers with high biological and disease specificities. The unique biophysical properties of protein stereo‐folding and proteostasis are abundant and detectable during the early phases of diseases, subtype‐classified in protein biology, flexible in multiple function measures, central in network regulations, important in phase separation biology, and predictable using computational biology.

In conclusion, protein stereo‐folding and proteostasis are important components of the intra‐ and extracellular microenvironment that maintain the optimal bio‐ecosystem for cell and organelle interactions. With AI‐based system analyses and virtual screening, efficient and accurate protein structure prediction modelling will accelerate translational progress from protein biology to drug discovery, from protein stereo‐folding to novel target models, and from proteostasis to next generation biomarkers. This convergence of foundational protein biology and AI‐accelerated analyses forms the three essential pillars for advancing data‐driven, personalised clinical precision medicine (Figure 1D). In addition, the functions of proteostasis vary according to local microenvironmental components, properties, and activities during development and in response to pathogens, therapies, or functional changes. Computational modelling of protein stereo‐folding and structure provides new insights into understanding the nature of proteostasis and is expected to integrate the stereo structure of protein with the spatiotemporal distributions and interactions of proteins for large‐scale discovery, analysis and design. A deeper understanding of protein stereo‐folding and proteostasis depends highly on advancing our knowledge of structural biology, quality of data curation, accuracy of model architecture, repeatability of large‐scale pre‐training, and improvement of interpretability methods. Thus, clinical and translational medicine is entering a new era of drug and biomarker discovery, as well as clinical precision medicine, based on protein spatiotemporal structure, location, and homeostasis.

## AUTHOR CONTRIBUTIONS

Yuhan Wang and Yuyang Qiu contributed to the literature preparation and manuscript writing. Xiangdong Wang was responsible for manuscript writing and discussion.

## CONFLICT OF INTEREST STATEMENT

The authors declare no conflicts of interest.

## FUNDING INFORMATION

The funding from Zhongshan Hospital of Fudan University, China for Shanghai Institute of Clinical Bioinformatics.

## ETHICAL APPROVAL

Not applicable.

## Data Availability

Not applicable.
